# Bis(2,2′-bipyridine-κ^2^ *N*,*N*′)dibromido­cadmium(II)

**DOI:** 10.1107/S1600536809042597

**Published:** 2009-10-23

**Authors:** Bi-Song Zhang

**Affiliations:** a College of Materials Science and Chemical Engineering, Jinhua College of Profession and Technology, Jinhua, Zhejiang 321017, People’s Republic of China

## Abstract

In the title complex mol­ecule, [CdBr_2_(C_10_H_8_N_2_)_2_], the Cd^II^ ion is six-coordinated by two *cis*-arranged bromide anions and four N atoms of two bidentate 2,2′-bipyridine ligands in a distorted octa­hedral geometry. The dihedral angle formed by the mean planes through the bipyridine ligands is 87.01 (11)°. In the crystal packing, π–π stacking inter­actions [centroid–centroid distances = 3.837 (6) and 3.867 (11) Å] link adjacent complex mol­ecules into chains running parallel to the *b* axis. The chains are further connected by inter­molecular C—H⋯Br hydrogen bonds into a three-dimensional network.

## Related literature

For the crystal structure of the isostructural manganese(II) derivative, see: Hwang & Ha (2007 ▶).
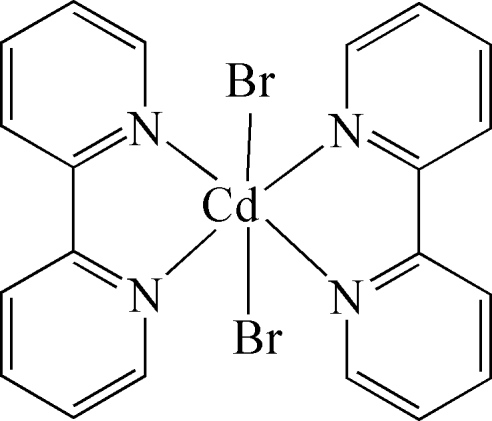


## Experimental

### 

#### Crystal data

[CdBr_2_(C_10_H_8_N_2_)_2_]
*M* *_r_* = 584.59
Monoclinic, 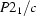

*a* = 8.9105 (18) Å
*b* = 14.446 (3) Å
*c* = 16.039 (3) Å
β = 98.66 (3)°
*V* = 2041.0 (7) Å^3^
*Z* = 4
Mo *K*α radiation
μ = 5.00 mm^−1^
*T* = 290 K
0.10 × 0.10 × 0.10 mm

#### Data collection

Rigaku R-AXIS RAPID diffractometer
Absorption correction: multi-scan (*ABSCOR*; Higashi, 1995 ▶) *T*_min_ = 0.605, *T*_max_ = 0.611
18624 measured reflections
4632 independent reflections
3399 reflections with *I* > 2σ(*I*)
*R*_int_ = 0.050

#### Refinement

*R*[*F* ^2^ > 2σ(*F* ^2^)] = 0.038
*wR*(*F* ^2^) = 0.136
*S* = 1.14
4632 reflections
244 parameters
H-atom parameters constrained
Δρ_max_ = 0.89 e Å^−3^
Δρ_min_ = −0.83 e Å^−3^

### 

Data collection: *RAPID-AUTO* (Rigaku, 1998 ▶); cell refinement: *RAPID-AUTO*; data reduction: *CrystalStructure* (Rigaku/MSC, 2002 ▶); program(s) used to solve structure: *SHELXS97* (Sheldrick, 2008 ▶); program(s) used to refine structure: *SHELXL97* (Sheldrick, 2008 ▶); molecular graphics: *SHELXTL* (Sheldrick, 2008 ▶); software used to prepare material for publication: *SHELXL97*

## Supplementary Material

Crystal structure: contains datablocks I, global. DOI: 10.1107/S1600536809042597/rz2371sup1.cif

Structure factors: contains datablocks I. DOI: 10.1107/S1600536809042597/rz2371Isup2.hkl

Additional supplementary materials:  crystallographic information; 3D view; checkCIF report

## Figures and Tables

**Table 1:** Hydrogen-bond geometry (Å, °)

*D*—H⋯*A*	*D*—H	H⋯*A*	*D*⋯*A*	*D*—H⋯*A*
C3—H3⋯Br2^i^	0.93	2.95	3.748 (8)	144
C7—H7⋯Br2^ii^	0.93	2.98	3.708 (5)	136
C17—H17⋯Br1^iii^	0.93	2.94	3.710 (6)	141
C18—H18⋯Br2^iv^	0.93	2.93	3.624 (7)	133

Symmetry codes: (i) 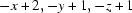; (ii) 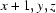; (iii) 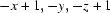; (iv) 
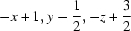
.

**Fig. 1.**
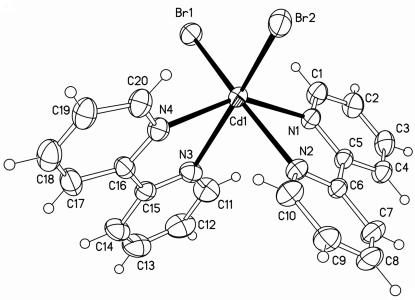
The molecular structure of the title compound. Displacement ellipsoids are drawn at the 50% probability level.

**Fig. 2.**
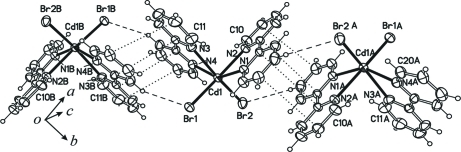
Partial packing diagram of the title compound showing intermolecular H bonds and π—π stacking interactions as dashed lines. Displacement ellipsoids are drawn at the 50% probability level. Symmetry codes: (A) 2-x, 1-y, 1-z; (B) 1-x, -y, 1-z.

## supplementary crystallographic information

### Comment

In the course of our studies aimed at the synthesis of new cadmium(II) 2-bromobenzoato complexes, the title compound was obtained accidentally, 2-bromobenzoic acid acting as a source for bromide anions. Hereafter, the crystal structure of the unexpected product obtained is reported.

The title compound is isostructural with the corresponding manganese(II) complex (Hwang & Ha, 2007). The cadmium(II) metal atom is is six-coordinated by two *cis*-arranged bromide anions and four N atoms of two bidentate 2,2'-bipyridine ligands in a distorted octahedral geometry (Fig. 1). The bipyridine ligands are not strictly planar  the dihedral angle between adjacent pyridine rings being 7.59 (16) and 4.90 (18)°. The dihedral angle formed by the mean planes through the bipyridine ligands is 87.01 (11)°. In the crystal packing, complex molecules are  linked into chains parallel to the *b* axis by π—π stacking interactions, with centroid-to-centroid distances of 3.837 (6) and 3.867 (11) Å (Fig. 2). The chains are further connected by intermolecular C—H···Br hydrogen bonds into a three-dimensional network (Table 1).

### Experimental

To a water solution of CdCl_2_. (0.27 g, 1.47 mmol) was added a 1M solution of Na_2_CO_3_.The CdCO_3_ precipitate was separated by filtration and washed with distilled water. The freshly prepared CdCO_3_, 2,2'-bipyridine (0.08 g, 0.51 mmol), 2-bromobenzoic acid (0.05 g, 0.24 mmol) in CH_3_OH/H_2_O (1:2 v/v; 15 ml) were mixed and stirred for 2 h. The resulting suspension was then heated in a 23 ml Teflon-lined stainless steel autoclave at 433 K for 5800 minutes. After cooling to room temperature, the solid formed was filtered off. The resulting filtrate was allowed to stand at room temperature, and evaporation for 4 months afforded red single crystals suitable for X-ray analysis.

### Refinement

H atoms on C atoms were positioned geometrically and refined as riding atoms, with C—H = 0.93 Å and with *U*_iso_(H) = 1.2*U*_eq_(C).

### Crystal data

**Table:**

[CdBr_2_(C_10_H_8_N_2_)_2_]	*F*(000) = 1128
*M**_r_* = 584.59	*D*_x_ = 1.902 Mg m^−^^3^
Monoclinic, *P*2_1_/*c*	Mo *K*α radiation, λ = 0.71073 Å
Hall symbol: -P 2ybc	Cell parameters from 13300 reflections
*a* = 8.9105 (18) Å	θ = 3.1–27.5°
*b* = 14.446 (3) Å	µ = 5.00 mm^−^^1^
*c* = 16.039 (3) Å	*T* = 290 K
β = 98.66 (3)°	Block, red
*V* = 2041.0 (7) Å^3^	0.10 × 0.10 × 0.10 mm
*Z* = 4	

### Data collection

**Table:**

Rigaku R-AXIS RAPID diffractometer	4632 independent reflections
Radiation source: fine-focus sealed tube	3399 reflections with *I* > 2σ(*I*)
graphite	*R*_int_ = 0.050
ω scans	θ_max_ = 27.5°, θ_min_ = 3.1°
Absorption correction: multi-scan (*ABSCOR*; Higashi, 1995)	*h* = −11→10
*T*_min_ = 0.605, *T*_max_ = 0.611	*k* = −18→18
18624 measured reflections	*l* = −20→19

### Refinement

**Table:**

Refinement on *F*^2^	Primary atom site location: structure-invariant direct methods
Least-squares matrix: full	Secondary atom site location: difference Fourier map
*R*[*F*^2^ > 2σ(*F*^2^)] = 0.038	Hydrogen site location: inferred from neighbouring sites
*wR*(*F*^2^) = 0.136	H-atom parameters constrained
*S* = 1.14	*w* = 1/[σ^2^(*F*_o_^2^) + (0.0714*P*)^2^] where *P* = (*F*_o_^2^ + 2*F*_c_^2^)/3
4632 reflections	(Δ/σ)_max_ < 0.001
244 parameters	Δρ_max_ = 0.89 e Å^−^^3^
0 restraints	Δρ_min_ = −0.83 e Å^−^^3^

### Special details

**Table:**

Geometry. All esds (except the esd in the dihedral angle between two l.s. planes) are estimated using the full covariance matrix. The cell esds are taken into account individually in the estimation of esds in distances, angles and torsion angles; correlations between esds in cell parameters are only used when they are defined by crystal symmetry. An approximate (isotropic) treatment of cell esds is used for estimating esds involving l.s. planes.
Refinement. Refinement of F^2^ against ALL reflections. The weighted R-factor wR and goodness of fit S are based on F^2^, conventional R-factors R are based on F, with F set to zero for negative F^2^. The threshold expression of F^2^ > 2sigma(F^2^) is used only for calculating R-factors(gt) etc. and is not relevant to the choice of reflections for refinement. R-factors based on F^2^ are statistically about twice as large as those based on F, and R- factors based on ALL data will be even larger.

### Fractional atomic coordinates and isotropic or equivalent isotropic displacement parameters (Å^2^)

**Table:**

	*x*	*y*	*z*	*U*_iso_*/*U*_eq_	
Cd1	0.69224 (3)	0.27479 (2)	0.53117 (2)	0.04035 (14)	
N1	0.8618 (4)	0.3596 (3)	0.4587 (3)	0.0457 (9)	
N2	0.9463 (4)	0.2806 (3)	0.6104 (3)	0.0461 (10)	
N3	0.7832 (5)	0.1333 (3)	0.4672 (3)	0.0516 (10)	
N4	0.6434 (4)	0.1378 (3)	0.6057 (3)	0.0479 (10)	
Br1	0.45650 (6)	0.27703 (4)	0.40944 (4)	0.0621 (2)	
Br2	0.60886 (6)	0.39268 (5)	0.64099 (4)	0.0712 (2)	
C1	0.8134 (6)	0.4015 (4)	0.3861 (4)	0.0573 (14)	
H1	0.7102	0.3994	0.3653	0.069*	
C2	0.9077 (6)	0.4480 (4)	0.3397 (4)	0.0637 (15)	
H2	0.8698	0.4761	0.2887	0.076*	
C3	1.0589 (7)	0.4515 (4)	0.3713 (4)	0.0669 (16)	
H3	1.1258	0.4825	0.3418	0.080*	
C4	1.1125 (6)	0.4090 (4)	0.4471 (4)	0.0548 (13)	
H4	1.2153	0.4110	0.4688	0.066*	
C5	1.0102 (5)	0.3630 (3)	0.4904 (3)	0.0430 (11)	
C6	1.0568 (5)	0.3196 (3)	0.5738 (3)	0.0426 (11)	
C7	1.2045 (5)	0.3221 (4)	0.6161 (4)	0.0611 (15)	
H7	1.2814	0.3469	0.5897	0.073*	
C8	1.2375 (6)	0.2884 (4)	0.6963 (4)	0.0682 (18)	
H8	1.3364	0.2905	0.7247	0.082*	
C9	1.1227 (6)	0.2511 (4)	0.7349 (4)	0.0624 (15)	
H9	1.1415	0.2295	0.7901	0.075*	
C10	0.9787 (6)	0.2470 (5)	0.6885 (4)	0.0574 (14)	
H10	0.9013	0.2197	0.7128	0.069*	
C11	0.8526 (6)	0.1343 (5)	0.3996 (4)	0.0669 (16)	
H11	0.8733	0.1912	0.3765	0.080*	
C12	0.8956 (7)	0.0543 (6)	0.3618 (4)	0.082 (2)	
H12	0.9446	0.0575	0.3146	0.098*	
C13	0.8648 (8)	−0.0290 (6)	0.3951 (5)	0.086 (2)	
H13	0.8923	−0.0838	0.3710	0.103*	
C14	0.7932 (6)	−0.0306 (5)	0.4638 (4)	0.0657 (16)	
H14	0.7713	−0.0870	0.4872	0.079*	
C15	0.7520 (5)	0.0523 (4)	0.5001 (3)	0.0478 (12)	
C16	0.6702 (5)	0.0545 (4)	0.5747 (3)	0.0475 (12)	
C17	0.6210 (6)	−0.0250 (4)	0.6101 (4)	0.0662 (16)	
H17	0.6405	−0.0829	0.5886	0.079*	
C18	0.5426 (7)	−0.0178 (5)	0.6777 (4)	0.0719 (18)	
H18	0.5076	−0.0711	0.7011	0.086*	
C19	0.5158 (6)	0.0670 (5)	0.7108 (4)	0.0620 (15)	
H19	0.4640	0.0733	0.7566	0.074*	
C20	0.5694 (6)	0.1418 (4)	0.6725 (3)	0.0569 (14)	
H20	0.5534	0.2000	0.6943	0.068*	

### Atomic displacement parameters (Å^2^)

**Table:**

	*U*^11^	*U*^22^	*U*^33^	*U*^12^	*U*^13^	*U*^23^
Cd1	0.0351 (2)	0.0449 (3)	0.0407 (2)	−0.00392 (12)	0.00467 (14)	−0.00158 (14)
N1	0.0384 (18)	0.051 (3)	0.047 (2)	−0.0046 (17)	0.0033 (16)	0.003 (2)
N2	0.038 (2)	0.054 (3)	0.044 (2)	−0.0011 (17)	0.0001 (17)	−0.0011 (19)
N3	0.055 (2)	0.051 (3)	0.048 (3)	0.0066 (19)	0.0068 (18)	−0.005 (2)
N4	0.047 (2)	0.050 (3)	0.045 (2)	−0.0040 (18)	0.0053 (17)	−0.002 (2)
Br1	0.0497 (3)	0.0604 (4)	0.0685 (4)	−0.0015 (2)	−0.0160 (3)	−0.0041 (3)
Br2	0.0621 (4)	0.0684 (4)	0.0902 (5)	−0.0174 (3)	0.0342 (3)	−0.0351 (3)
C1	0.053 (3)	0.065 (4)	0.052 (3)	−0.009 (3)	0.003 (2)	0.011 (3)
C2	0.072 (4)	0.066 (4)	0.053 (3)	−0.011 (3)	0.010 (3)	0.013 (3)
C3	0.071 (4)	0.065 (4)	0.070 (4)	−0.021 (3)	0.027 (3)	0.000 (3)
C4	0.045 (3)	0.060 (4)	0.061 (4)	−0.008 (2)	0.015 (2)	−0.002 (3)
C5	0.037 (2)	0.045 (3)	0.047 (3)	−0.0046 (19)	0.0102 (19)	−0.006 (2)
C6	0.034 (2)	0.045 (3)	0.049 (3)	−0.0020 (19)	0.0075 (18)	−0.007 (2)
C7	0.037 (2)	0.074 (4)	0.070 (4)	−0.001 (2)	−0.001 (2)	0.000 (3)
C8	0.045 (3)	0.076 (5)	0.075 (4)	0.005 (3)	−0.017 (3)	−0.009 (3)
C9	0.061 (3)	0.065 (4)	0.055 (4)	0.005 (3)	−0.013 (3)	0.001 (3)
C10	0.050 (3)	0.076 (4)	0.046 (3)	−0.001 (3)	0.005 (2)	0.003 (3)
C11	0.079 (4)	0.074 (4)	0.052 (4)	0.007 (3)	0.026 (3)	−0.006 (3)
C12	0.077 (4)	0.106 (6)	0.065 (4)	0.018 (4)	0.022 (3)	−0.030 (4)
C13	0.085 (4)	0.074 (5)	0.094 (6)	0.018 (4)	0.001 (4)	−0.037 (4)
C14	0.073 (4)	0.049 (4)	0.072 (4)	0.010 (3)	−0.001 (3)	−0.019 (3)
C15	0.041 (2)	0.047 (3)	0.050 (3)	0.002 (2)	−0.007 (2)	−0.006 (2)
C16	0.050 (3)	0.039 (3)	0.048 (3)	−0.002 (2)	−0.008 (2)	−0.001 (2)
C17	0.073 (4)	0.045 (4)	0.074 (4)	−0.010 (3)	−0.009 (3)	0.010 (3)
C18	0.070 (4)	0.077 (5)	0.065 (4)	−0.018 (3)	−0.001 (3)	0.023 (4)
C19	0.063 (3)	0.073 (4)	0.050 (3)	−0.017 (3)	0.008 (2)	0.008 (3)
C20	0.056 (3)	0.065 (4)	0.051 (3)	−0.012 (3)	0.013 (2)	0.000 (3)

### Geometric parameters (Å, °)

**Table:**

Cd1—N1	2.380 (4)	C7—C8	1.365 (9)
Cd1—N4	2.385 (4)	C7—H7	0.9300
Cd1—N2	2.425 (4)	C8—C9	1.382 (9)
Cd1—N3	2.478 (4)	C8—H8	0.9300
Cd1—Br2	2.6360 (8)	C9—C10	1.384 (7)
Cd1—Br1	2.6440 (11)	C9—H9	0.9300
N1—C1	1.326 (7)	C10—H10	0.9300
N1—C5	1.345 (6)	C11—C12	1.386 (9)
N2—C10	1.334 (7)	C11—H11	0.9300
N2—C6	1.343 (6)	C12—C13	1.362 (11)
N3—C11	1.328 (6)	C12—H12	0.9300
N3—C15	1.329 (7)	C13—C14	1.355 (9)
N4—C16	1.337 (7)	C13—H13	0.9300
N4—C20	1.341 (7)	C14—C15	1.404 (7)
C1—C2	1.378 (8)	C14—H14	0.9300
C1—H1	0.9300	C15—C16	1.492 (8)
C2—C3	1.367 (8)	C16—C17	1.383 (8)
C2—H2	0.9300	C17—C18	1.379 (9)
C3—C4	1.381 (8)	C17—H17	0.9300
C3—H3	0.9300	C18—C19	1.371 (9)
C4—C5	1.395 (7)	C18—H18	0.9300
C4—H4	0.9300	C19—C20	1.363 (8)
C5—C6	1.478 (7)	C19—H19	0.9300
C6—C7	1.388 (6)	C20—H20	0.9300
			
N1—Cd1—N4	148.10 (14)	C7—C6—C5	123.2 (4)
N1—Cd1—N2	68.09 (14)	C8—C7—C6	120.4 (5)
N4—Cd1—N2	89.58 (14)	C8—C7—H7	119.8
N1—Cd1—N3	87.20 (15)	C6—C7—H7	119.8
N4—Cd1—N3	67.59 (15)	C7—C8—C9	119.5 (5)
N2—Cd1—N3	84.70 (14)	C7—C8—H8	120.3
N1—Cd1—Br2	104.92 (11)	C9—C8—H8	120.3
N4—Cd1—Br2	96.31 (10)	C8—C9—C10	117.7 (6)
N2—Cd1—Br2	87.84 (10)	C8—C9—H9	121.2
N3—Cd1—Br2	162.24 (11)	C10—C9—H9	121.2
N1—Cd1—Br1	97.18 (9)	N2—C10—C9	122.8 (5)
N4—Cd1—Br1	101.28 (9)	N2—C10—H10	118.6
N2—Cd1—Br1	164.09 (11)	C9—C10—H10	118.6
N3—Cd1—Br1	88.71 (10)	N3—C11—C12	122.8 (7)
Br2—Cd1—Br1	102.30 (3)	N3—C11—H11	118.6
C1—N1—C5	119.1 (4)	C12—C11—H11	118.6
C1—N1—Cd1	121.2 (3)	C13—C12—C11	118.7 (6)
C5—N1—Cd1	119.6 (3)	C13—C12—H12	120.7
C10—N2—C6	119.6 (4)	C11—C12—H12	120.7
C10—N2—Cd1	122.2 (3)	C14—C13—C12	118.8 (6)
C6—N2—Cd1	118.3 (3)	C14—C13—H13	120.6
C11—N3—C15	119.0 (5)	C12—C13—H13	120.6
C11—N3—Cd1	123.4 (4)	C13—C14—C15	120.5 (7)
C15—N3—Cd1	117.4 (3)	C13—C14—H14	119.8
C16—N4—C20	118.2 (5)	C15—C14—H14	119.8
C16—N4—Cd1	120.2 (3)	N3—C15—C14	120.2 (5)
C20—N4—Cd1	120.9 (4)	N3—C15—C16	117.1 (4)
N1—C1—C2	123.5 (5)	C14—C15—C16	122.7 (5)
N1—C1—H1	118.2	N4—C16—C17	120.6 (6)
C2—C1—H1	118.2	N4—C16—C15	117.0 (5)
C3—C2—C1	117.7 (6)	C17—C16—C15	122.4 (5)
C3—C2—H2	121.1	C18—C17—C16	119.3 (6)
C1—C2—H2	121.1	C18—C17—H17	120.3
C2—C3—C4	120.1 (5)	C16—C17—H17	120.3
C2—C3—H3	120.0	C19—C18—C17	120.7 (6)
C4—C3—H3	120.0	C19—C18—H18	119.7
C3—C4—C5	119.0 (5)	C17—C18—H18	119.7
C3—C4—H4	120.5	C20—C19—C18	116.2 (6)
C5—C4—H4	120.5	C20—C19—H19	121.9
N1—C5—C4	120.5 (5)	C18—C19—H19	121.9
N1—C5—C6	116.9 (4)	N4—C20—C19	125.0 (6)
C4—C5—C6	122.5 (4)	N4—C20—H20	117.5
N2—C6—C7	120.0 (5)	C19—C20—H20	117.5
N2—C6—C5	116.7 (4)		

### Hydrogen-bond geometry (Å, °)

**Table:**

*D*—H···*A*	*D*—H	H···*A*	*D*···*A*	*D*—H···*A*
C3—H3···Br2^i^	0.93	2.95	3.748 (8)	144
C7—H7···Br2^ii^	0.93	2.98	3.708 (5)	136
C17—H17···Br1^iii^	0.93	2.94	3.710 (6)	141
C18—H18···Br2^iv^	0.93	2.93	3.624 (7)	133

Symmetry codes: (i) −*x*+2, −*y*+1, −*z*+1; (ii) *x*+1, *y*, *z*; (iii) −*x*+1, −*y*, −*z*+1; (iv) −*x*+1, *y*−1/2, −*z*+3/2.
